# Comparison of Side Effects Between Miniscrew-Assisted Rapid Palatal Expansion (MARPE) and Surgically Assisted Rapid Palatal Expansion (SARPE) in Adult Patients: A Scoping Review

**DOI:** 10.3390/dj13020047

**Published:** 2025-01-22

**Authors:** Nicolò Sicca, Giulia Benedetti, Agnese Nieri, Sara Vitale, Gaia Lopponi, Silvia Mura, Alessio Verdecchia, Enrico Spinas

**Affiliations:** Department of Surgical Sciences, Postgraduate School in Orthodontics, University of Cagliari, 09124 Cagliari, Italy; n.sicca@studenti.unica.it (N.S.); g.benedetti@studenti.unica.it (G.B.); a.nieri@studenti.unica.it (A.N.); s.vitale6@studenti.unica.it (S.V.); g.lopponi@studenti.unica.it (G.L.); silviamura3@gmail.com (S.M.)

**Keywords:** MARPE, SARPE, maxillary expansion, complications, side effects, adverse effects

## Abstract

**Background/Objectives**: The aim of this study is to investigate the side effects of two techniques of rapid maxillary expansion—Surgically Assisted Rapid Palatal Expansion (SARPE) and Miniscrew-Assisted Rapid Palatal Expansion (MARPE)—in adult patients, to guide the selection of the most cost-effective clinical treatment plan. **Methods**: The review protocol was developed in accordance with the Preferred Reporting Items for Systematic Reviews and Meta-Analysis—extension for Scoping Reviews (PRISMA-ScR) guidelines. Eligibility criteria were defined based on the study objectives. The research team formulated a PICO question to identify relevant studies in the literature. Five databases were queried: MEDLINE (via PubMed), Scopus, Cochrane Library, Web of Science, and Embase. Additionally, a manual search was conducted. **Results**: The computer-assisted search identified 746 articles, of which only 26 fully met the inclusion criteria and were included in the scoping review. Among the included studies, 11 were retrospective, 12 were prospective, and 3 were randomized clinical trials. SARPE was evaluated in 21 studies, MARPE in 4 studies, and 1 article reported complications associated with both techniques. The side effects described in the studies were synthesized and categorized into five groups: expansion failure, asymmetric expansion, dentoalveolar issues, surgical complications, and appliance-related problems. **Conclusions**: The results indicate that both techniques involve risks. The most reported adverse effects were dentoalveolar and surgical in nature. Dentoalveolar side effects, such as dental tipping, were predominantly associated with the MARPE technique, while surgical complications were more commonly observed with the SARPE technique. Patient age is crucial for treatment choice as well as proper design and planning of the expansion device. Consequently, careful patient selection and treatment planning are essential to minimize the side effects of maxillary expansion in adult patients.

## 1. Introduction

Transverse maxillary deficiency is a malocclusion with a prevalence of 21% in children and approximately 10% in adults [1]. Its treatment involves increasing the transverse width of the maxilla by separating the midpalatal suture [2].

Rapid maxillary expansion (RME) is the most widely used and accepted technique for maxillary expansion in children. This method employs natural teeth as anchorage to transmit mechanical forces; however, in skeletally mature patients, conventional RME yields limited skeletal effects due to increased interdigitation of the midpalatal suture and adjacent articulations. Traditional RME may also result in adverse dentoskeletal effects such as buccal tipping [3], gingival recession, fenestrations [4], and root resorption [5] of the supporting teeth.

In individuals over 18 years of age, the likelihood of complete midpalatal suture maturation, classified as stages D and E according to Angelieri et al. [6,7,8], is significantly higher. Consequently, beyond this age, traditional RME relying solely on dental anchorage is contraindicated.

Surgically Assisted Rapid Palatal Expansion (SARPE) was introduced to address the limitations of traditional rapid maxillary expansion by incorporating osteotomy and expansion appliances [9]. SARPE is an osteogenic separation procedure comprising four phases: the surgical phase (osteotomy), a latency phase, an osseous distraction phase, and a consolidation phase. Notably, the surgical component of SARPE lacks a standardized protocol, and no universally accepted “gold standard” technique has been established [10].

Over time, various SARPE approaches have been developed, each employing different osteotomy techniques to reduce resistance and facilitate separation of the palatal suture. Commonly described techniques include an osteotomy extending from the piriform aperture to the maxillary tuberosity [11], or one beginning at the nasofrontal suture and extending to the maxillary tuberosity [12]. Other approaches involve modifications of a Le Fort I osteotomy with an additional midpalatal suture osteotomy [13].

Recent advancements have focused on less invasive techniques with reduced surgical interventions, such as those proposed by Morselli [14] and Lindorf [15], which involve selective weakening of facial complex sutures to enable maxillary expansion. These techniques have further evolved to include minimally invasive approaches that can be performed under local anesthesia [16].

The devices used for post-operative skeletal expansion have also undergone significant advancements. Initially, the transpalatal distractor (TPD), introduced by Mommaerts in 1999 [17], utilized two telescopic cylinders with skeletal anchorage. Since then, more modern expanders have been developed, including tooth-borne devices (anchored to teeth), bone-borne devices (anchored via miniscrews), and hybrid systems combining dental and skeletal anchorage through miniscrews (tooth-bone-borne expanders).

SARPE is generally regarded as a low-risk procedure; however, several serious complications have been reported in the literature. These include life-threatening epistaxis, cerebrovascular accidents, skull base fractures with reversible oculomotor nerve palsy, and orbital compartment syndrome [18,19,20]. Milder complications include postoperative hemorrhage, pain, sinusitis, irritation or ulceration of the palatal tissue, asymmetrical expansion, nasal septum deviation, periodontal issues, and relapse [21,22,23].

Miniscrew-Assisted Rapid Palatal Expansion (MARPE) has emerged as a significant alternative to surgical intervention for correcting transverse maxillary deficiency in adults. This technique utilizes four implants inserted through the double cortical bone of the hard palate and palatal bone to generate orthopedic forces [24], promoting parallel expansion of the midpalatal suture [25]. Compared to conventional rapid maxillary expansion, MARPE is associated with reduced biological and financial costs and fewer dentoalveolar side effects [26]. However, MARPE does not eliminate dental side effects, as root volume loss and dental tipping of the anchorage teeth have been documented [27].

Despite its advantages, MARPE may pose challenges in patients over 18 years of age, with a notable risk of suture non-opening, reported in 14–16% of cases [24,26].

The aim of the scoping review is to investigate the side effects of each treatment procedure in order to guide the choice of the best cost-effective clinical treatment plan for adult patients.

## 2. Materials and Methods

### 2.1. Protocol and Registration

This scoping review was drafted using the Preferred Reporting Items for Systematic reviews and Meta-Analysis extension for Scoping Reviews (PRISMA-ScR) guidelines [28,29]. The protocol of this study was not registered.

The research team developed a PICO question to find relevant studies in bibliography:

- Population: adult patients in permanent dentition diagnosed with a transverse maxillary discrepancy;
- Intervention: MARPE technique;
- Comparison: SARPE technique;
- Outcome: side effects of each treatment procedure.

### 2.2. Eligibility Criteria

The eligibility criteria were determined according to the study goals.

Papers on the side effects of transverse expansion of the palate with any type of MARPE or SARPE technique and appliance design were selected.

One of the following adverse effects should be mentioned in the research: expansion failure, asymmetric expansion, dentoalveolar effects (dental tipping, tooth necrosis, tooth discoloration, tooth mobility, root resorption, periodontal issues), surgical complications (pain, discomfort, bleeding, swelling, edema, hematoma, infection, dehiscence, nerve injury, mucosal damage, nasal septum deviation, lacrimation), or appliance-related issues (appliance failure, distortion, mobility, or loss of TADs).

Only patients over 18 years of age, with permanent dentition and no history of systemic diseases or craniofacial syndromes, were included. Studies involving patients with periodontal disease or a history of surgical palatal expansion or other types of maxillofacial surgeries were excluded.

Accepted study designs encompassed randomized clinical trials and prospective or retrospective observational studies, while meta-analysis, reviews, in vitro or finite elements studies, animal studies, case series and case reports were excluded. For inclusion, articles needed to have the full text, without restriction on the publication year.

**Figure 1:**
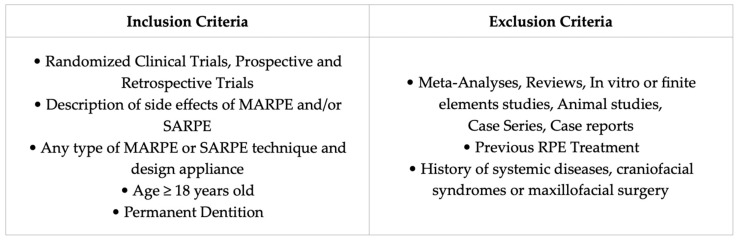
The figure outlines the inclusion and exclusion criteria applied to select articles for the literature review.

The eligibility criteria were summarized in Figure 1.

### 2.3. Information Sources and Search Strategy

The search was performed between September and November 2024 across five databases: MEDLINE (via PubMed), Scopus, Cochrane Library, Web of Science, and Embase. The initial search strategy was developed for PubMed and subsequently tailored to the other databases. The final search was conducted on 30 November 2024 and studies published before 1 December 2024 were considered eligible, with no restrictions on the year of publication.

An electronic search of the grey literature was also carried out using OpenGrey.

Details of the complete electronic search strategy are provided in Table 1.

### 2.4. Selection of Sources of Evidence

Four reviewers (N.S., A.N., S.V., and G.B.) independently screened the search results. Duplicate records were removed using Zotero and manually verified for accuracy. An Excel sheet containing all eligibility criteria was prepared to streamline the selection process. Titles and abstracts were evaluated against these criteria, and full-text versions of relevant articles were retrieved for thorough assessment. Following the full-text review, the final set of studies for inclusion was determined.

Any disagreements among the reviewers were resolved through consultation with an expert (E.S.).

### 2.5. Data Charting Process

The four reviewers together developed a second Excel chart including all the variables to extract: first author, year of publication, country, study design, sample size, mean age, data collection, type of expansion, type of expander, expansion protocol, and side effects.

Complications were classified into five categories: failure of expansion, asymmetric expansion, dentoalveolar, surgical, and appliance-related side effects.

All the authors charted the data, discussed the results, and improved the selection in a dynamic way.

### 2.6. Synthesis of Results

The studies were divided into two groups according to the expansion technique performed—MARPE or SARPE. The side effects were separately analyzed and summarized based on the above-mentioned categories.

## 3. Results

### 3.1. Selection of Sources of Evidence

The computer-assisted search identified 744 articles across six databases: PubMed (*n =* 235), Scopus (*n =* 149), Cochrane Library (*n =* 71), Web of Science (*n =* 50), Embase (*n =* 239), and OpenGrey (*n =* 0). Additionally, two studies were retrieved through manual searching. After removing duplicates (*n =* 191), 555 articles remained for screening.

Following an evaluation of titles and abstracts, 416 studies were excluded either because they were not pertinent or for the article type. The full texts of five articles could not be retrieved. Of the 134 studies assessed in full text, 108 were excluded for not meeting the inclusion criteria: 21 due to article type (FEM analysis and animal studies), 6 because the sample included craniofacial syndromes, 64 due to the sample falling outside the age range, and 17 because adverse effects were not described.

**Figure 2:**
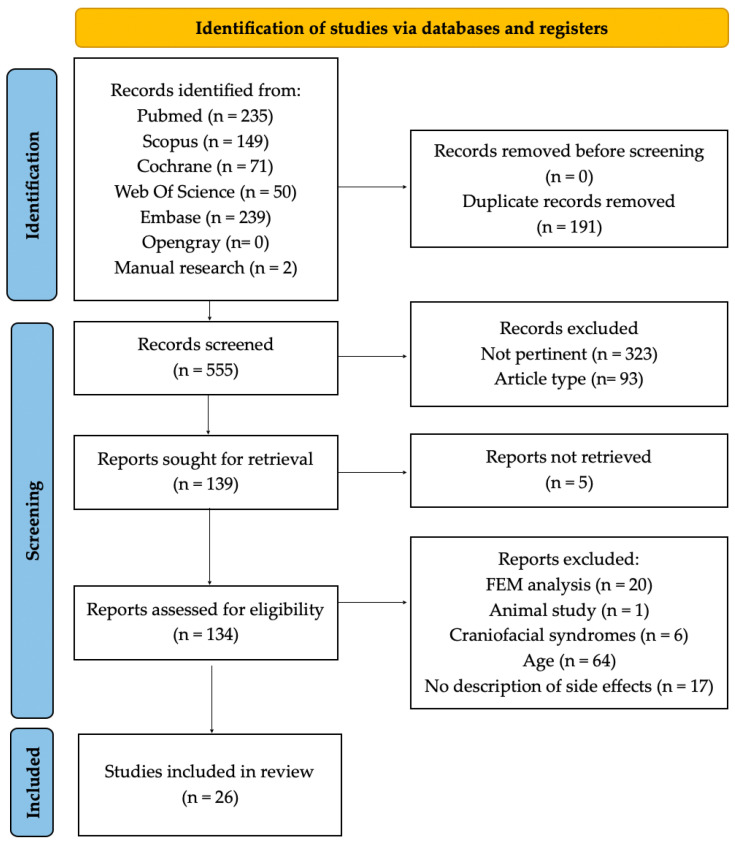
The figure shows the flowchart used for the study selection process, according to the Preferred Reporting Items for Systematic reviews and Meta-analysis extension for Scoping Reviews (PRISMA-ScR) guidelines.

Ultimately, 26 studies [30,31,32,33,34,35,36,37,38,39,40,41,42,43,44,45,46,47,48,49,50,51,52,53,54,55] satisfied the inclusion criteria and were included in the scoping review. The details of the literature search and selection procedure are shown in the flowchart in Figure 2.

### 3.2. Characteristics of Sources of Evidence

The main characteristics of the included studies [30,31,32,33,34,35,36,37,38,39,40,41,42,43,44,45,46,47,48,49,50,51,52,53,54,55] are summarized in Table 2; Table 3 to provide a comprehensive overview. The data were divided into two separate tables to enhance clarity and ease of consultation.

The studies were conducted in various countries: Austria (*n =* 2) [31,54], Belgium (*n =* 1) [50], Brazil (*n =* 5) [34,38,44,47,49], China (*n =* 1) [52], Egypt (*n =* 1) [35], France (*n =* 1) [42], Germany (*n =* 2) [45,48], India (*n =* 1) [32], Israel (*n =* 1) [46], Italy (*n =* 1) [30], Republic of Korea (*n =* 2) [33,43], Russia (*n =* 1) [36], Spain (*n =* 1) [37], Syria (*n =* 1) [31], Turkey (*n =* 4) [39,40,41,51], and the USA (*n =* 2) [53,55].

Among the 26 included studies, 11 were retrospective [30,34,36,43,45,48,50,51,53,54,55], 12 were prospective [31,35,37,38,39,40,42,44,46,47,49,52], and 3 [32,33,41] were randomized clinical trials. Sample sizes ranged from 8 patients [37] to 665 patients [36], with a mean age varying between 30 and 34 years.

Dentoalveolar side effects were primarily assessed using CBCT (*n =* 15) [32,33,35,36,38,40,41,42,43,48,50,51,52,54,55] or periapical or occlusal x-rays (*n =* 5) [33,34,44,45,47] and measurements on study models (*n =* 10) [31,32,34,35,37,38,39,42,44,45]. Surgical side effects and appliance-related issues were evaluated through clinical examinations (*n =* 13) [32,34,37,39,42,44,45,46,47,49,50,53,54] and, in some cases, patient questionnaires focused on pain and discomfort (*n =* 2) [47,55]. Additional evaluation methods included photographs (*n =* 4) [32,35,38,40], cephalograms (*n =* 7) [32,34,35,37,38,39,44], and orthopantomograms (*n =* 3) [32,37,44]. One study employed superficial electromyography (sEMG) to assess changes in masticatory muscle activity following palatal expansion [30], while another article used polysomnography to analyze patients with obstructive sleep apnea syndrome (OSAS) [55].

Details of the expansion type and protocol are provided in Table 3. Most studies focused on the SARPE technique (*n =* 21) [30,31,34,35,36,37,38,39,40,41,42,44,45,46,47,48,49,50,51,53,55], while four investigated MARPE [32,33,43,52]. One article examined both techniques, reporting that patients underwent SARPE following MARPE failure [54].

The expanders used were categorized based on anchorage type: tooth-borne appliances (*n =* 18) [30,31,34,35,38,40,41,42,44,45,46,47,48,49,50,51,52,53], bone-borne appliances (*n =* 10) [32,36,37,39,42,45,48,50,53,54], and hybrid appliances combining tooth- and bone-borne anchorage (*n =* 7) [32,33,37,41,42,43,55]. Comparative evaluations of different appliance types were performed in nine studies [32,37,41,42,44,45,48,50,53].

In some trials, the appliance was activated intraoperatively to assess its effectiveness (*n =* 10) [31,32,35,38,41,42,44,47,48,51]. Daily activations ranged from 0.2 mm to 1 mm. For SARPE, many clinicians implemented a latency period between the surgical procedure and the start of appliance activation (*n =* 19) [30,31,34,35,36,37,38,39,40,42,44,45,47,48,50,51,53,54,55].

The specific expansion protocols for all studies are detailed in Table 3.

### 3.3. Results of Individual Sources of Evidence

Side effects reported in the included studies were summarized in Table 4.

They were divided into five categories: failure of expansion, asymmetric expansion, dentoalveolar, surgical, and appliance-related issues.

#### 3.3.1. Failure of Expansion

Failure of midpalatal suture separation was reported in 4 out of 22 articles [36,42,47,53] for the SARPE group and in 3 out of 5 studies [33,43,54] for the MARPE group. Across these papers, the failure rate ranged from 2% to 29%. Instances such as insufficient expansion, relapse, or inability to achieve the planned diastema were also classified as failure.

#### 3.3.2. Asymmetric Expansion

Asymmetric expansion was seen in seven studies [36,42,44,45,50,53,55], all of which utilized the SARPE technique.

#### 3.3.3. Dentoalveolar Side Effects

Dentoalveolar side effects were documented in 21 out of 26 trials [32,33,34,35,36,38,39,40,41,42,43,44,45,46,48,49,50,51,52,53,55]. The most common complications were periodontal issues, including gingival recessions (*n =* 7) [34,36,42,46,49,50,53], alveolar bone loss (*n =* 8) [32,36,40,41,43,46,52,53], and periodontal attachment loss (*n =* 3) [45,49,55].

Dental tipping during expansion was reported in eight studies [32,33,40,41,43,48,51,52], four of which investigated MARPE [32,33,43,52]. Winsauer et al. [54] was the only MARPE study that did not report tipping of the anchoring teeth. In some cases, dental tipping was associated with bending of the supporting alveolar bone (*n =* 2) [39,51].

Pulp sensitivity impairment, tooth necrosis, and/or tooth discoloration were observed in eight studies [35,36,39,42,44,45,53,55], with none involving MARPE. In most cases, these problems were resolved through root canal treatment, though Williams et al. [53] described two cases requiring tooth extraction.

Root resorption of varying severity was reported in two studies [41,45], affecting either the anchorage teeth or anterior maxillary teeth near the interdental osteotomy site.

Tooth mobility, observed in three studies [38,45,50], was consistently a transient phenomenon.

#### 3.3.4. Surgical Complications

Surgical complications were predominantly observed in studies employing the SARPE technique, except for Choi et al. [33], who documented a case of nasal mucosal inflammation following MARPE intervention as the miniscrew penetrated the nasal floor.

Pain emerged as the most commonly reported surgical complication. (*n =* 6) [34,38,39,44,47,50], occurring either postoperatively or during the expansion phase. This symptom was alleviated through modifications to the activation protocol, analgesic administration, or removal of the expander to enhance palatal mucosal hygiene. Additionally, two studies [35,47] noted patient discomfort during expander activation.

Palatal mucosal trauma caused by the expansion appliance during activation resulted in inflammation, erosions, ulcers, sloughing, fistulas, and, in severe cases, mucosal necrosis (*n =* 5) [36,37,42,53,55].

Bleeding was a frequently reported surgical complication (*n =* 4) [36,39,50,53], originating from either the palatal mucosa or as epistaxis. Nasal complications included mucosal thickening reported by Choi et al. [33], as well as maxillary sinus perforation and infection described by Drobyshev et al. [36] and Williams et al. [53], respectively.

Hematoma (*n =* 2) [30,53], edema (*n =* 3) [35,38,47], and swelling (*n =* 2) [30,31] were identified as minor surgical sequelae that often resolved spontaneously. Williams et al. [53] also reported an instance of subcutaneous emphysema.

Neurosensory disturbances (e.g., paresthesia or hypoesthesia) were documented in four studies [36,50,53,55], affecting the maxillary division of the trigeminal nerve (V2), infraorbital nerve branches, and nasopalatine nerve. These complications were exclusively associated with SARPE procedures. Similarly, occurrences of local infection, wound dehiscence, and lacrimation were linked solely to the SARPE technique (*n =* 6) [34,39,44,50,53,55].

#### 3.3.5. Appliance-Related Issues

Eight studies documented appliance-related complications associated with both MARPE and SARPE techniques [33,34,36,39,42,45,50,54]. Leyder et al. [42] and Winsauer et al. [54] reported screw deformation during MARPE procedures. Similarly, Ploder et al. [45] observed screw loosening and fracture in 13% of the study sample. Winsauer et al. [54] also described one instance of abutment loss during the retention period and two additional cases of screw deformation. Other technical issues involved the expander itself, including deformation, displacement, and loss of the distractor (*n =* 4) [34,36,39,50].

### 3.4. Synthesis of the Results

There were great differences in methodology among the included studies, such as device design, expansion protocol, measurement, and factors that may affect the results.

The most observed side effects were dentoalveolar and surgical in nature. Most of the dental side effects, such as dental tipping, were observed with the MARPE technique, while surgical complications predominantly with the SARPE technique.

## 4. Discussion

### 4.1. Summary of Evidence

The purpose of this review is to describe the side effects of two different maxillary expansion techniques, MARPE and SARPE, in adult patients.

The side effects described in the 26 selected articles were divided into five categories: expansion failure, asymmetric expansion, dentoalveolar, surgical and appliance-related issues. Dentoalveolar complications are the most reported adverse effects and occur with both MARPE and SARPE techniques. While these side effects cannot be eliminated, their severity varies depending on the technique employed. Expansion failure and asymmetric expansion represent significant clinical challenges, often requiring a second intervention, which prolongs treatment duration and increase the biological burden on the patient. These complications are strongly influenced by the patient’s age, skeletal maturity, and the expansion method used. Surgical complications are predominantly associated with the SARPE technique and the invasiveness of the surgical approach. Additionally, the design of the appliance is directly linked not only to dentoalveolar complications but also to device-related issues.

Dental tipping is one of the most reported adverse effects, equally associated with MARPE and SARPE techniques [32,33,40,41,43,48,51,52]. This phenomenon appears to be an inherent characteristic of maxillary expansion methods, to the extent that it may not qualify as a true side effect but rather as a transient condition that resolves naturally over time.

In the study by Lim et al. [43], which examines dentoskeletal effects one year after MARPE expansion, a recurrence of buccal inclination in the dental elements was observed, surpassing the relapse noted in the alveolar segments containing the teeth. This finding indicates a more pronounced skeletal expansion component during the follow-up compared to the immediate post-expansion phase.

Similarly, Sygouros et al. [51] characterize dental tipping as an intrinsic outcome of skeletal expansion that tends to improve spontaneously during the retention phase.

In several studies included in this review, dental tipping was not reported, likely because the majority utilized the SARPE technique. Notably, Basu et al. [32] found significantly greater dental tipping in patients treated with the traditional MARPE approach compared to those undergoing midpalatal suture weakening through corticopuncture. Similarly, Karabiber et al. [40] observed that a unilateral osteotomy resulted in reduced dental tipping and less alveolar bone loss on the osteotomy side compared to the contralateral side. This difference may stem from the initial force being transmitted predominantly to the teeth before the suture opens, a process mitigated by the median palatal osteotomy in SARPE or suture weakening via corticopuncture in MARPE procedures.

Dental tipping has been reported in patients treated with both tooth-borne and bone-borne devices, though its frequency appears influenced by appliance design. Ning et al. [56] demonstrated a statistically significant difference in tipping between tooth-bone-borne and purely bone-borne devices. Similarly, Cozzani et al. [57] highlighted greater stress on anchorage teeth with tooth-borne appliances, leading to tipping, a phenomenon absents with bone-borne expanders. Lin et al. [58] further noted reduced alveolar bone loss and diminished dental tipping in the first premolars of patients using bone-borne expanders compared to those with tooth-borne devices.

More severe dental complications, such as tooth necrosis, discoloration, tooth loss, and mobility, were reported in studies that employed the SARPE technique for maxillary expansion [36,38,39,42,44,45,50,53,55]. Remarkably, in all cases, the surgical approach included palatal suture osteotomy. Four studies additionally performed pterygomaxillary disjunction (PMD) [44,45,50,53], while one study incorporated down fracture following a Le Fort I osteotomy [42]. These findings suggest a correlation between the increasing complexity of the surgical procedures used in SARPE and a higher risk of adverse effects on dental structures.

Periodontal issues were reported in nine studies [34,36,41,42,43,45,46,49,50], with the most frequently observed adverse effects being gingival recession, increased probing depth (PD), and clinical attachment loss (CAL). It is noteworthy that all these studies used expansion devices with dental anchorage, except for the study by Drobyshev et al. [36], which used a bone-borne device and found gingival recessions in 0.7% of patients. This highlights that dental anchorage devices can complicate the maintenance of adequate oral hygiene, leading to increased inflammation in the teeth serving as anchors. These findings are consistent with the conclusions of the meta-analysis by Bi et al. [59], which states that the type of expander and the type of anchorage should be chosen based on clinical conditions, while accounting for potential adverse dental effects when selecting a hybrid or purely dental anchorage.

Root resorption was also noted in two studies [41,45], both of which utilized dental anchorage devices. This complication is less common in adult patients undergoing expansion with skeletal anchorage supported by mini implants compared to traditional dental anchorage [60]. Nonetheless, root resorption remains a recognized complication associated with maxillary expansion, irrespective of the device or technique employed [61].

Asymmetric expansion, observed in both SARPE and MARPE, typically manifests in the anteroposterior plane with a pyramidal pattern. This is attributed to increased resistance in the posterior region, including the pterygomaxillary suture and zygomatic buttress, as well as the location of the center of rotation of the zygomaticomaxillary complex just above the frontozygomatic suture [62]. However, asymmetry can also occur in the transverse plane, with one side expanding more than the other.

Asymmetric expansion appears to be closely linked to the surgical method and often necessitates additional surgical interventions for correction, resulting in significant patient discomfort [63]. The mean rate of asymmetric expansion with SARPE in this review was 8.5%, higher than the 4.4% reported by Carvalho et al. [64]. Carvalho’s study identified factors such as the absence of PMD and a slow activation protocol as contributing to asymmetric expansion. However, this review found no consistent correlation, as asymmetry was also described in studies utilizing rapid activation protocols and PMD. These findings underscore the need for further high-quality research to elucidate the factors most strongly associated with asymmetric expansion in SARPE procedures.

Although it has not been described in the articles included in this review, cases of asymmetric expansion with the MARPE technique have been reported in the literature. Kim et al. [65] demonstrated that this complication may arise from an asymmetric fracture of the circummaxillary suture system rather than solely from uneven opening of the palatal midline suture. Additionally, patients with frontal plane asymmetries, such as a deviated chin, appear more prone to this issue, which may worsen or become less predictable after MARPE therapy. In Kim et al.’s study, the incidence of asymmetric expansion was 30% and was characterized as difficult to predict [65].

Failure or inadequate outcomes in maxillary expansion were reported in eight of the included studies [33,36,42,43,47,53,54,55]. Among these, two studies specifically investigated the MARPE technique, reporting failure rates of 16% [33] and 17% [43]. These rates are consistent with success rates documented in the literature, which range from 84% to 88% [66,67]. A plausible explanation for these findings is the predominant focus of MARPE studies on young adult populations. Success rates decline significantly with advancing age, dropping to approximately 20% in patients aged 30–37 years, who commonly exhibit midpalatal suture maturation stages D or E [68].

Yoon et al. [67] corroborated these observations, demonstrating that 68% of patients with suture-opening failure were over 25 years old. In such cases, combining MARPE with corticopuncture to weaken the suture prior to expansion has been shown to improve outcomes. Similarly, the study by Winsauer et al. [54] reported a 15% failure rate with the MARPE technique, with a need for SARPE in patients who experienced failure of expansion assisted by mini implants; interestingly, the mean age of patients experiencing non-surgical expansion failure in this study was 41.3 years, further supporting the inverse relationship between age and MARPE success rates.

In contrast, studies examining SARPE generally report lower failure rates compared to MARPE. An exception is the study by Sant’Ana et al. [47], which documented a 29% failure rate in the subset of patients who did not undergo palatal suture osteotomy. These findings suggest that SARPE is a more favorable approach in adult patients with midpalatal suture maturation stages D or E [8].

For example, the Distraction Osteogenesis Maxillary Expansion (DOME), as described by Yoon et al. [55], integrates the use of the Maxillary Skeletal Expander (MSE) with the surgical approach of SARPE, omitting pterygomaxillary disjunction. This technique demonstrated a 100% success rate with a low complication rate in a patient cohort with a mean age of 30 years. Additionally, the study reported a reduction in the Apnea-Hypopnea Index (AHI), indicating significant improvements in Obstructive Sleep Apnea (OSA) and associated symptoms, such as daytime sleepiness, consistent with findings from other studies [33].

Recently, minimally invasive techniques have been proposed to enhance the success rates of MARPE. Haas Junior et al. [21] introduced a SARPE protocol performed under local anesthesia within 19 min, combined with a bone-borne expander. This approach excludes PMD, which, according to Sangsari et al. [69], is not essential for achieving palatal suture expansion. This modification reduces operative time and postoperative complications, offering a more comfortable recovery for patients. This technique appears particularly advantageous for adult patients, who often face higher failure rates with MARPE. However, the study by Haas Junior et al. is limited by its small sample size and lack of long-term follow-up data to validate the procedure’s effectiveness.

Surgical complications are predominantly reported in studies employing the SARPE technique [30,31,33,34,35,36,37,38,39,42,44,47,50,53,54]. These adverse effects range from minor issues, such as epistaxis, swelling, edema, and hematomas—typically resolving without long-term consequences—to more severe outcomes, including paresthesia [36,53,55], palatal mucosal necrosis [53], and wound dehiscence [34,39]. The only exception is noted in the study by Choi et al. [33], which reported a case of nasal mucosal thickening following MARPE as the miniscrew penetrated the nasal floor.

According to the review by Carvalho et al. [64], the primary surgical complications in SARPE, though infrequent, were observed in studies both with and without PMD. This aligns with the findings of the present analysis, indicating that the occurrence of surgical complications cannot be reliably linked to a specific procedural approach. However, it is evident that higher surgical invasiveness is associated with an increased risk of major complications.

Further high-quality studies are necessary to identify the optimal surgical technique for SARPE, one that ensures safe execution while minimizing the risk of complications.

The highest rates of device failure were associated with bone-borne transpalatal distractors (TPDs) [36,39,45,50], which are considered particularly challenging to manage clinically. In other studies, the primary issues involved deformation or failure of the mini implants. Yoon et al. [67] attributed the loss of mini implants primarily to tissue inflammation surrounding the screws and to inadequate oral hygiene. Similarly, Bud et al. [70] identified mucosal inflammation, hyperplasia around the mini implants, and loosening or deformation of the screws as the most common complications associated with the MARPE technique.

The impingement of mini implants or device arms on the palatal mucosa is a significant contributor to inflammation, increasing the risk of screw failure. To mitigate these risks, maintaining a 1 mm clearance between the screw and the palate and a 3–4 mm clearance between the device’s side arms and the palatal shelf is recommended. Notably, inflammation often arises during the retention phase, likely due to the relapse tendency of the expanded hemipalate, while the screw width remains unchanged [71].

The review of the included articles highlights the significant influence of appliance design on the occurrence of side effects. Transpalatal devices (TPDs) are particularly challenging in terms of clinical management and are associated with a high failure rate. Tooth-borne devices have a negative impact on the anchoring elements, increasing the risk of root resorption, dental tipping, and periodontal issues. In contrast, bone-borne appliances supported by miniscrews, while still presenting instances of dental and alveolar tipping, appear to pose fewer risks to dental structures. Therefore, the precise selection of the appliance is crucial and should consider the patient’s characteristics in terms of periodontal health, dental condition, and ability to maintain adequate oral hygiene.

This study emphasizes that both MARPE and SARPE techniques in adult patients involve risks. From a clinical standpoint, thorough patient assessment is essential to determine the most appropriate technique. The patient’s age is the primary factor to consider. Young adult patients under 25 years of age demonstrate a high success rate in suture opening with the MARPE technique [72], making it a preferred approach as it is more conservative and does not require surgery with associated potential complications. Conversely, in patients over 25 years of age, the success rate of suture opening declines significantly, with studies reporting a success rate of 20% in individuals over 30 [68].

Some efforts to address this limitation, such as increasing the expander’s anchorage with additional miniscrews [73], have been explored. However, these modifications increase invasiveness and the risk of device-related complications, without ensuring consistent clinical success. Skeletal maturity, closely tied to age, is another critical consideration and can be evaluated using CBCT imaging of the palatal suture. In patients at stages D and E, the success rate of suture opening with MARPE is reduced, and a combined expansion and surgical approach is recommended.

Consistent with existing literature [72], this review also confirms that patient gender does not influence the success rate of MARPE expansion. From a cost–benefit perspective, in adult patients with skeletal maturity at stages D or E, techniques involving surgical weakening of the suture should be prioritized to prevent reoperation in case of MARPE failure. Furthermore, such patients are more prone to dental and periodontal complications. In contrast, for young adult patients with skeletal maturity at stages A, B, or C, the use of MARPE may be advantageous due to its lower biological cost, as it eliminates the need for extensive surgical intervention.

### 4.2. Future Directions

Future research aimed at improving treatment options for adult patients with maxillary transverse deficiency should focus on integrating minimally invasive surgical techniques in association with skeletal anchorage expanders. This combined approach has the potential to enhance therapeutic success rates while minimizing complications traditionally associated with surgical interventions.

To achieve this, it will be essential to develop a standardized surgical technique, to establish a clear distraction protocol, and to design an optimized appliance incorporating skeletal anchorage. Studies should specifically target adult patients with a maturation stage of the midpalatal suture classified as D or E [8] and include long-term follow-up to evaluate the stability and durability of the achieved outcomes.

Due to the considerable heterogeneity of the expansion protocols used, it was not possible to establish a direct correlation between the reported side effects and the specific expansion protocol employed. Although existing literature suggests an increased risk of asymmetric expansion with slow expansion protocols [64], this was not corroborated by the articles included in this review, where asymmetric expansion was observed in both rapid and slow expansion protocols. A comprehensive analysis comparing the influence of different expansion protocols on the occurrence of specific side effects represents a critical direction for future research.

### 4.3. Limitations

To the best of our knowledge, this study is the first to directly compare the complications associated with the two primary skeletal expansion techniques in adults.

The major limitation of the review lies in the significant heterogeneity among the included studies, particularly regarding the expansion activation protocols and the methods used to analyze complications.

Another key limitation is that the clinical trial team varies depending on the technique employed. In fact, for the SARPE technique, most of the studies fall under the expertise of maxillofacial surgery, whereas for the MARPE technique, the studies are predominantly within the field of dentistry. This disciplinary divide influences both the areas of focus and the types of complications reported, making direct comparisons challenging.

Ultimately, it is essential to remember that this research does not include a qualitative analysis of the studies, as it is not a systematic literature review [74]. Therefore, the obtained results should be interpreted with caution.

To address these issues, future research should adopt standardized protocols and involve more homogeneous, interdisciplinary research teams. Such efforts are essential to provide a clearer and more comprehensive understanding of the potential complications associated with these two expansion techniques.

## 5. Conclusions

The aim of this review was to evaluate the adverse effects of the two main palatal expansion techniques in adult patients to assist clinicians in the choice of the most cost-effective and clinically appropriate treatment plan.

Considering the limitations related to the heterogeneity and the absence of a qualitative assessment of the included studies, the obtained results allow for the following conclusions:

- Patient age is a critical determinant of the success rate in maxillary expansion. As age increases, the likelihood of requiring SARPE rather than MARPE rises, making SARPE essential for successful outcomes in older patients.
- Weakening the midpalatal suture through corticopunctures, combined with MARPE or SARPE, significantly reduces buccal inclination of the dental elements. However, this inclination often improves spontaneously during the retention phase.
- Severe dental complications associated with SARPE are infrequent but correlate with the invasiveness of the surgical technique employed.
- Dentoalveolar complications can be minimized by selecting expansion devices that avoid dental anchorage.
- Proper design and planning of the appliance are crucial to prevent tissue inflammation, which remains the leading cause of mini implant failure.

Finally, clinicians should be fully aware of the potential complications associated with both techniques and ensure patients are adequately informed about risks and alternative treatments. In this context, the study by Yoon et al. [67] provides a comprehensive informed consent model for MARPE; however, no equivalent model currently exists in the literature for SARPE. Future research should focus on developing standardized protocols for maxillary expansion that integrate minimally invasive surgical techniques with skeletal anchorage systems.

## Figures and Tables

**Table 1:** The table provides a detailed overview of the search strategies employed for the five databases consulted: MEDLINE (via PubMed), Scopus, Cochrane, Web of Science, and Embase.

Database	Search Strategy	Number of Results
MEDLINE (via PubMed)	(((bone screws[MeSH Terms])OR ((screw*[Title/Abstract])AND (bone[Title/Abstract]))OR (bone-anchored[Title/Abstract])OR (bone-borne[Title/Abstract])OR (implant anchorage*[Title/Abstract])OR (implant-supported[Title/Abstract])OR (miniimplant*[Title/Abstract])OR (micro implant*[Title/Abstract])OR (micro screw*[Title/Abstract])OR (mini implant*[Title/Abstract])OR (mini screw*[Title/Abstract])OR (mini-implant*[Title/Abstract])OR (miniscrew*[Title/Abstract])OR (mini-screw*[Title/Abstract])OR (orthodontic anchorage*[Title/Abstract])OR (orthodontic anchorage procedure*[Title/Abstract])OR (orthodontic anchorage technique*[Title/Abstract])OR (orthodontic anchoring procedure*[Title/Abstract])OR (skeletal anchorage*[Title/Abstract])OR (anchorage screw*[Title/Abstract])OR (temporary anchorage device*[Title/Abstract])OR ((anchorage procedure*[Title/Abstract])AND (orthodontic[Title/Abstract]))OR ((anchorage technique*[Title/Abstract])AND (orthodontic[Title/Abstract]))OR ((procedure*[Title/Abstract])AND (orthodontic anchorage*[Title/Abstract]))OR ((technique*[Title/Abstract])AND (orthodontic anchorage*[Title/Abstract]))OR (TAD[Title/Abstract])OR (TADs[Title/Abstract]))AND ((palatal expansion technique*[MeSH Terms])OR (palatal expansion technic*[Title/Abstract])OR (palatal expander*[Title/Abstract])OR (palatal expansion*[Title/Abstract])OR (maxilla expansion*[Title/Abstract])OR (maxillary expansion*[Title/Abstract])OR (maxillary suture expansion*[Title/Abstract])OR ((expansion*[Title/Abstract])AND (maxillary[Title/Abstract]))OR ((expansion technic*[Title/Abstract])AND (palatal[Title/Abstract]))OR ((expansion technique*[Title/Abstract])AND (palatal[Title/Abstract]))OR ((technic*[Title/Abstract])AND (palatal expansion*[Title/Abstract]))OR ((technique*[Title/Abstract])AND (palatal expansion*[Title/Abstract]))))OR (((MARPE[Title/Abstract])OR (MARME[Title/Abstract])))	235
Scopus	((TITLE-ABS-KEY (marpe) OR TITLE-ABS-KEY (marme))) OR (((TITLE-ABS-KEY (“bone screws”)) OR (TITLE-ABS-KEY (screw* AND bone)) OR (TITLE-ABS-KEY (bone-anchored)) OR (TITLE-ABS-KEY (bone-borne)) OR (TITLE-ABS-KEY (implant AND anchorage*)) OR (TITLE-ABS-KEY (implant-supported)) OR (TITLE-ABS-KEY (miniimplant*)) OR (TITLE-ABS-KEY (micro AND implant*)) OR (TITLE-ABS-KEY (micro AND screw*)) OR (TITLE-ABS-KEY (mini AND implant*)) OR (TITLE-ABS-KEY (mini AND screw*)) OR (TITLE-ABS-KEY (mini-implant*)) OR (TITLE-ABS-KEY (miniscrew*)) OR (TITLE-ABS-KEY (mini-screw*)) OR (TITLE-ABS-KEY (orthodontic AND anchorage*)) OR (TITLE-ABS-KEY (“orthodontic anchorage procedure*”)) OR (TITLE-ABS-KEY (“orthodontic anchorage technique*”)) OR (TITLE-ABS-KEY (“orthodontic anchoring procedure*”)) OR (TITLE-ABS-KEY (“orthodontic anchoring procedure*”)) OR (TITLE-ABS-KEY (skeletal AND anchorage*)) OR (TITLE-ABS-KEY (anchorage AND screw*)) OR (TITLE-ABS-KEY (temporary AND anchorage AND device*)) OR (TITLE-ABS-KEY (“anchorage procedure*” orthodontic)) OR (TITLE-ABS-KEY (“anchorage technique*” orthodontic)) OR (TITLE-ABS-KEY (procedure* “orthodontic anchorage*”)) OR (TITLE-ABS-KEY (technique* “orthodontic anchorage*”)) OR (TITLE-ABS-KEY (tad)) OR (TITLE-ABS-KEY (tads))) AND ((TITLE-ABS-KEY (“palatal expansion technique*”)) OR (TITLE-ABS-KEY (palatal AND expansion AND technic*)) OR (TITLE-ABS-KEY (palatal AND expander*)) OR (TITLE-ABS-KEY (palatal AND expansion*)) OR (TITLE-ABS-KEY (maxilla AND expansion*)) OR (TITLE-ABS-KEY (maxillary AND suture AND expansion*)) OR (TITLE-ABS-KEY (expansion* AND maxillary)) OR (TITLE-ABS-KEY (“expansion technic*” palatal)) OR (TITLE-ABS-KEY (“expansion technique*” palatal)) OR (TITLE-ABS-KEY (technic* “palatal expansion*”)) OR (TITLE-ABS-KEY (technique* “palatal expansion*”))))	149
Cochrane Library	#1 MeSH descriptor: [Palatal Expansion Technique] explode all trees #2 Palatal Expansion* Technique* #3 Expansion* Technic* Palatal #4 Expansion* Maxillary #5 Maxilla* Expansion* #6 Maxillary suture expansion* #7 palatal expander* #8 palatal expansion* #9 #1 OR #2 OR #3 OR #4 OR #5 OR #6 OR #7 OR #8 #10 anchorage procedure* orthodontic #11 anchorage screw* #12 anchorage technique* orthodontic #13 bone screw* #14 bone-anchored #15 bone-borne #16 miniimplant* #17 implant anchorage* #18 implant-supported #19 micro implant* #20 micro screw* #21 mini implant* #22 mini screw* #23 mini-implant* #24 miniscrew* #25 mini-screw* #26 orthodontic anchorage* #27 orthodontic anchorage procedure* #28 orthodontic anchorage technique* #29 orthodontic anchoring procedure* #30 screw* bone #31 skeletal anchorage* #32 tad #33 tads #34 temporary anchorage device* #35 #10 OR #11 OR # 12 OR #13 OR #14 OR #15 OR #16 OR #17 OR #18 OR #19 OR #20 OR #21 OR #22 OR #23 OR #24 OR #25 OR #26 OR #27 OR #28 OR #29 OR #30 OR #31 OR #32 OR #33 OR #34 #36 #9 AND #35 #37 MARPE #38 MARME #39 #36 OR #37 OR #38	71
Web of Science	#1 ((((((((((((((((((((((((((TS=(bone screws))OR TS=(screw* bone))OR TS=(bone-anchored))OR TS=(bone-borne))OR TS=(implant anchorage*))OR TS=(implant-supported))OR TS=(miniimplant*))OR TS=(micro implant*))OR TS=(micro screw*))OR TS=(mini implant*))OR TS=(mini screw*))OR TS=(mini-implant*))OR TS=(miniscrew*))OR TS=(mini-screw*))OR TS=(orthodontic anchorage*))OR TS=(“orthodontic anchorage procedure*”))OR TS=(“orthodontic anchorage technique*”))OR TS=(“orthodontic anchoring procedure*”))OR TS=(skeletal anchorage*))OR TS=(anchorage screw*))OR TS=(“temporary anchorage device*”))OR TS=(“anchorage procedure*” orthodontic))OR TS=(“anchorage technique*” orthodontic))OR TS=(procedure* “orthodontic anchorage*”))OR TS=(technique* “orthodontic anchorage*”))OR TS=(TAD))OR TS=(TADs) #2 ((((((((((TS=(“palatal expansion technique*”))OR TS=(palatal expansion technic*))OR TS=(palatal expander*))OR TS=(palatal expansion*))OR TS=(maxilla expansion*))OR TS=(maxillary expansion*))OR TS=(“maxillary suture expansion*”))OR TS=(“expansion technic*” palatal))OR TS=(“expansion technique*” palatal))OR TS=(technic* “palatal expansion*”))OR TS=(technique* “palatal expansion*”) #3 (TS=(MARPE))OR TS=(MARME) #4 #2 AND #1 #5 #4 OR #3	50
Embase	‘marme’:ti, ab, kw OR ‘marpe’:ti, ab, kw OR ((‘palatal expansion’/exp OR (‘expansion technique*’:ti, ab, kw AND palatal:ti, ab, kw)OR ‘palatal expansion technique*’:ti, ab, kw OR (technique*:ti, ab, kw AND ‘palatal expansion*’:ti, ab, kw)OR ‘palatal expansion technic*’:ti, ab, kw OR (‘expansion technic*’:ti, ab, kw AND palatal:ti, ab, kw)OR ‘palatal expansion technic*’:ti, ab, kw OR (technic*:ti, ab, kw AND ‘palatal expansion*’:ti, ab, kw)OR ‘maxillary expansion*’:ti, ab, kw OR (expansion*:ti, ab, kw AND maxillary:ti, ab, kw)OR ‘palatal expansion technique*’:ti, ab, kw OR ‘palatal expansion*’:ti, ab, kw OR ‘palatal expander*’:ti, ab, kw OR ‘maxilla expansion*’:ti, ab, kw OR ‘maxillary suture expansion*’:ti, ab, kw AND (‘bone screw’/exp OR (screw*:ti, ab, kw AND bone:ti, ab, kw)OR miniscrew*:ti, ab, kw OR miniimplant*:ti, ab, kw OR ‘micro screw*’:ti, ab, kw OR ‘skeletal anchorage*’:ti, ab, kw OR tad:ti, ab, kw OR tads:ti, ab, kw OR ‘temporary anchorage device*’: ti, ab, kw OR ‘anchorage screw*’:ti, ab, kw OR ‘micro implant*’:ti, ab, kw OR ‘mini implant*’:ti, ab, kw OR ‘mini screw*’:ti, ab, kw OR ‘implant supported’:ti, ab, kw OR ‘implant anchorage*’:ti, ab, kw OR ‘orthodontic anchorage*’:ti, ab, kw OR ‘bone borne’:ti, ab, kw OR ’bone anchored’:ti, ab, kw OR ‘orthodontic anchorage procedure*’:ti, ab, kw OR ‘orthodontic anchoring procedure*’:ti, ab, kw OR ‘orthodontic anchorage technique*’:ti, ab, kw OR (technique*:ti, ab, kw AND ‘orthodontic anchorage’:ti, ab, kw)OR (‘anchorage technique*’:ti, ab, kw AND orthodontic:ti, ab, kw)OR (procedure*:ti, ab, kw AND ‘orthodontic anchorage’:ti, ab, kw)OR (‘anchorage procedure*’:ti, ab, kw AND orthodontic:ti, ab, kw))	239

**Table 2:** The table provides details (first author, publication year, study design, sample size, and mean age) about the 26 articles included in the scoping review.

First Author [Reference]	Year	Country	Study Design	Sample Size (n° of Patients)	Mean Age (Years)
Abate A. et al. [30]	2023	Italy	Retrospective study	20	27.3
Al-Ouf K. et al. [31]	2010	Austria, Syria	Prospective study	17	30.7
Basu S. et al. [32]	2023	India	Randomized Clinical Trial	18	20.8
Choi E.H.A. et al. [33]	2023	Republic of Korea	Randomized Clinical Trial	32	23
Contar C.M.M. et al. [34]	2009	Brazil	Retrospective study	14	33.5
**First Author [Reference]**	**Year**	**Country**	**Study Design**	**Sample Size (n° of Patients)**	**Mean Age (Years)**
Daif E.T. [35]	2014	Egypt	Prospective study	30	24
Drobyshev A. et al. [36]	2021	Russia	Retrospective study	665	25.3
Fernández Sanromán J. et al. [37]	2010	Spain	Prospective study	8	28.5
Goldenberg D.C. et al. [38]	2007	Brazil	Prospective study	15	24.5
Gunbay T. et al. [39]	2008	Turkey	Prospective study	10	22.3
Karabiber G. et al. [40]	2019	Turkey	Prospective study	16	18.4
Kayalar E. et al. [41]	2015	Turkey	Randomized Clinical Trial	20	19.4
Leyder P. et al. [42]	2018	France	Prospective study	55	23.6
Lim H. et al. [43]	2017	Republic of Korea	Retrospective study	29	21.6
Pereira M.D. et al. [44]	2017	Brazil	Prospective study	90	26.1
Ploder O. et al. [45]	2020	Germany	Retrospective study	54	28.8
Rachmiel A. et al. [46]	2020	Israel	Prospective study	32	19–54
Sant’Ana L.F.M. et al. [47]	2016	Brazil	Prospective study	24	24.29
Seeberger R. et al. [48]	2015	Germany	Retrospective study	33	26
Sendyk M. et al. [49]	2018	Brazil	Prospective study	17	25–45
Smeets M. et al. [50]	2019	Belgium	Retrospective study	111	26
Sygouros A. et al. [51]	2014	Turkey	Retrospective study	26	18.8
Wang C. et al. [52]	2023	China	Prospective study	40	22.42 ± 3.38
Williams B.J.D. et al. [53]	2012	USA	Retrospective Study	120	29.5 (22–39)
Winsauer H. et al. [54]	2021	Austria	Retrospective study	33	29.1 ± 10.2 (18–58)
Yoon A. et al. [55]	2020	USA	Retrospective study	75	30.5 ± 8.5

**Table 3:** The table deep dives into data collection methods, type of expansion technique, type of appliance used, and expansion protocols used in the included studies.

First Author and Year [Reference]	Data Collection	Intervention (Type of Expansion)	Intervention (Appliance Design)	Protocol of Expansion (Until the Planned/Desired Expansion Was Achieved)
Abate A. et al., 2023 [30]	EMG Examinations	SARPE (Le fort I osteotomy + midpalatal osteotomy)	Tooth-borne Hyrax-type expander	7-day latency period, then one activation (0.25 mm) twice daily
Al-Ouf K. et al., 2010 [31]	Study models	SARPE (Bilateral osteotomies on both sides of the midpalatal suture in the floor of the nasal cavity starting from the posterior border and continuing towards the anterior border of the piriform aperture)	Tooth-borne Hyrax-type expander	Four activations (0.25 mm each) intra-operatively; 7-day latency period. Daily activation protocol not specified
Basu S. et al., 2023 [32]	Clinical evaluation; introral and extraoral photographs; cephalograms; OPT; study models; CBCT measurements	MARPE (Group A: corticopuncture-facilitated BBRME Group B: conventional MARPE)	Group A: tooth-bone-borne Hyrax-type expander Group B: bone-borne	Both groups: one activation intra-operatively; two activations daily until the appearance of midline diastema, then one activation per day
Choi E.H.A. et al., 2023 [33]	CBCT measurements; periapical x-rays	MARPE	Tooth-bone-borne Hyrax-type expander	One activation (0.2 mm) daily
Contar C.M.M. et al., 2009 [34]	Clinical evaluation; study models; cephalograms; periapical x-rays	SARPE (modified Le Fort I osteotomy + midpalatal osteotomy)	Tooth-borne Hyrax-type expander	5-day latency period; two activations (0.25 mm) per day, one every 12 h
Daif E.T., 2014 [35]	Photographs, study models, cephalograms, CBCT measurements	SARPE (Bilateral zygomatic buttress osteotomy + midpalatal osteotomy)	Tooth-borne Hyrax-type expander	Eight activations (0.25 mm each) intraoperatively; 5-day latency period, then two activations per day
Drobyshev A. et al., 2021 [36]	CBCT measurements	SARPE (Le Fort I osteotomy + midpalatal osteotomy)	Bone-borne TPD	7-day latency period, then activations from 0.3 mm to 1 mm daily
**First Author and Year [Reference]**	**Data Collection**	**Intervention (Type of Expansion)**	**Intervention (Appliance Design)**	**Protocol of Expansion (Until the Planned/Desired Expansion Was Achieved)**
Fernández Sanromán J. et al., 2010 [37]	Clinical evaluation; OPT; cephalograms; study models	SARPE (Zygomaticomaxillary buttress osteotomy + midpalatal osteotomy)	Two Hyrax-type expanders: Bone-borne and tooth-bone-borne	7-day latency period; three activations (0.2 mm each) daily
Goldenberg D.C. et al., 2007 [38]	Photographs; study models; cephalograms; CBCT measurements	SARPE (modified Le Fort I osteotomy + midpalatal osteotomy)	Tooth-borne Hyrax-type expander	Four activations (0.25 mm each) intraoperatively; 3- day latency period, then two activations per day
Gunbay T. et al., 2008 [39]	Clinical evaluation; cephalograms; study models	SARPE (osteotomies of the anterior, lateral, and medial of the maxilla’s sutures)	Bone-borne TPD	7-day latency period; Five activations (0.2 mm each) per day
Karabiber G. et al., 2019 [40]	Intraoral and extraoral photographs; CBCT measurements	Unilateral SARPE (asymmetric anterior and lateral osteotomies + asymmetric PMD + midpalatal osteotomy)	Asymmetrically designed tooth-borne Hyrax-type expander	5-day latency period, then two activations (0.25 mm each) daily
Kayalar E. et al., 2015 [41]	CBCT measurements	SARPE (Le Fort I osteotomy + midpalatal osteotomy + PMD)	Tooth-borne Hyrax type expander; Tooth-bone-borne Hyrax-type expander	Intraoperative activation until a diastema of 1 mm was shown. Two activations (0.25 mm each) per day
Leyder P. et al., 2018 [42]	Clinical evaluation; CBCT measurements; study models	SARPE (Le Fort I osteotomy + down fracture + medial or single lateral corticotomy)	Three types of TPD: Tooth-borne (*n =* 36), bone-borne (*n =* 11), tooth-bone-borne (*n =* 8)	Intraoperative activation to achieve less than 3 mm osseous separation; 4-day latency period, then activation of 0.53 mm daily
Lim H., 2017 et al. [43]	CBCT measurements	MARPE	Tooth-bone-borne (modified) Hyrax-type expander	Two activations (0.2 mm each) per day
Pereira M.D. et al., 2017 [44]	Clinical evaluation; study models; cephalograms; OPT; periapical and occlusal x-rays;	SARPE (Le Fort I osteotomy + PMD)	Tooth-borne Haas- (*n =* 29) and Hyrax-(*n =* 61) type expanders	Eight activations (0.2 mm each) intraoperatively; 4-day latency period, then two activations per day
**First Author and Year [Reference]**	**Data Collection**	**Intervention (Type of Expansion)**	**Intervention (Appliance Design)**	**Protocol of Expansion (Until the Planned/Desired Expansion Was Achieved)**
Ploder O. et al., 2020 [45]	Clinical evaluation; radiographic evaluation; study models	SARPE (Le Fort I osteotomy + midpalatal osteotmy + PMD)	Tooth-borne splint-type appliance Bone-borne appliance (TPD device) Bone-borne appliance (OMI appliance)	Tooth-borne appliance: 6-day latency period, then three activations (0.2 mm each) per day TPD appliance: 4 to 6-day latency period, then two activations (0.5 mm each) per day OMI appliance: 5-day latency period, then three activations (0.17 mm each) per day
Rachmiel A. et al., 2020 [46]	Clinical evaluation	SARPE	Tooth-borne Hyrax-type expander	Two activations (0.25 mm each) per day
Sant’Ana L.F.M. et al., 2016 [47]	Clinical evaluation; occlusal radiographs; Pain questionnaire	SARPE (Group 1: partial bilateral maxillary antero-lateral ostoeotomies + midpalatal osteotomy; Group 2: bilateral maxillary antero-lateral ostoeotomies)	Tooth-borne Hyrax-type expander	Four activations (0.25 mm each) intraoperatively; 2-day latency period, then one activation twice a day
Seeberger R. et al., 2015 [48]	CBCT measurements	SARPE (Subtotal Le Fort I osteotomy + PMD)	Two types of device: tooth-borne Hyrax-type expander and bone-borne TPD	Tooth-borne group: Four activations (0.2 mm each) intraoperatively; 5 to 7-day latency period, then two activations per day; Bone-borne group: same protocol but each activation was 0.25 mm
Sendyk M. et al., 2018 [49]	Clinical evaluation	SARPE (Le fort I osteotomy + PMD + osteotomy of the anterior region of the maxilla)	Tooth-borne Hyrax-type expander	Two activations per day, one in the morning, and one at night
Smeets M. et al., 2019 [50]	Clinical evaluation; CBCT measurements	SARPE (Le Fort I osteotomy + PMD + midpalatal osteotomy)	Tooth-borne Hyrax-type expander + Bone-borne TPD expander	7-day latency period, then two activations (0.25 mm each) daily
**First Author and Year [Reference]**	**Data Collection**	**Intervention (Type of Expansion)**	**Intervention (Appliance Design)**	**Protocol of Expansion (Until the Planned/Desired Expansion Was Achieved)**
Sygouros A. et al. 2014 [51]	CBCT measurements	SARPE (Le Fort I+ 2 groups: SARPE with PMD and SARPE without PMD)	Tooth-borne Hyrax-type expander	Eight activations (0.25 mm each) intraoperatively; 3-day latency period, then two activations daily
Wang C. et al., 2023 [52]	CBCT measurements	MARPE	Tooth-borne Hyrax-type expander	Two activations (0.2 mm each) per day until a diastema was observed between the maxillary central incisors, then one activation daily
Williams B.J.D. et al., 2012 [53]	Clinical evaluation	SARPE (Le Fort I Osteotomy + Midpalatal osteotomy + interdental osteotomy + PMD)	Tooth-borne (*n =* 118) and bone-borne (*n =* 2) appliances	5 to 7-day latency period; two activations (0.25 mm each) per day
Winsauer H. et al., 2021 [54]	Clinical evaluation; CBCT measurements	MARPE Patients without visible diastema after 4 months underwent SARPE	Bone-borne MICRO-4 expander	MARPE Group: two activations (0.17 mm each) per day for the first week; then six activations and six deactivations daily, plus every third day, the device was additionally activated by 0.17 mm SARPE group: 5-day latency period, then three activations a day (0.5 mm daily)
Yoon A. et al., 2020 [55]	CBCT measurements; polysomnography; questionnaire	SARPE (Le Fort I + midpalatal osteotomy)	Tooth-bone-borne expander	5 to 7-day latency period, then one activation (0.25 mm) per day

CBCT, Cone beam computed tomography; EMG, Electromyographic; MARPE, Miniscrew-Assisted Rapid Palatal Expansion; OMI, Orthodontic mini-implant; OPT, Orthopantomography; PMD, Pterygomaxillary disjunction; SARPE, Surgically Assisted Rapid Palatal Expansion; TPD, Transpalatal distractor.

**Table 4:** The table summarizes adverse effects from included studies based on the type of expansion (SARPE or MARPE) and categorizes them by type. Incidence rates of complications are provided where available.

First Author, Year [Reference]	Intervention Type	Expansion Failure	Asymmetric Expansion	Dentoalveolar	Surgical	Appliance-Related Issues
Abate A. et al., 2023 [30]	SARPE	N/R	N/R	N/R	Hematoma (100%) Swelling (100%)	N/R
Al-Ouf K. et al., 2010 [31]	SARPE	N/R	N/R	N/R	Swelling	N/R
Basu S. et al., 2023 [32]	MARPE	N/R	N/R	Dental tipping (100% group B > group A); Buccal alveolar bone loss (100% group B > group A)	N/R	N/R
Choi E.H.A. et al., 2023 [33]	MARPE	16%	N/R	Dental tipping (100%)	Thickening of the nasal mucous membrane	Screw failure
Contar C.M.M. et al., 2009 [34]	SARPE	N/R	N/R	Gingival recession (14%)	Pain (14%) Wound dehiscence (14%)	Appliance deformation (7%)
Daif E.T., 2014 [35]	SARPE	N/R	N/R	Temporary impairment of the pulp sensitivity	Edema Discomfort	N/R
Drobyshev A. et al., 2021 [36]	SARPE	Insufficient expansion (5%) Relapse (3%)	4%	Gingival recession (0.7%) Tooth discoloration (0.5%) Alveolar bone loss (0.3%)	Paresthesia (30%) Palatal mucosa inflammation (9%) or necrosis (0.1%) Bleeding (1.1%) Maxillary sinus perforation (0.9%)	Distraction device displacement (9%) Distractor’s loss (3%)
**First Author, Year [Reference]**	**Intervention Type**	**Expansion Failure**	**Asymmetric Expansion**	**Dentoalveolar**	**Surgical**	**Appliance-Related Issues**
Fernández Sanromán J. et al., 2010 [37]	SARPE	N/R	N/R	N/R	Palatal mucosa inflammation (erosions, ulcers) (100%)	N/R
Goldenberg D.C. et al., 2007 [38]	SARPE	N/R	N/R	Tooth mobility (13%)	Pain (80%) Edema	N/R
Gunbay T. et al., 2008 [39]	SARPE	N/R	N/R	Tooth necrosis (20%) Buccal displacement of the left alveolar segment (10%)	Pain (30%) Nasal bleeding (20%) Wound dehiscence (20%) Inter-incisal septum fracture (20%)	Loosening of the distractor (20%)
Karabiber G. et al., 2019 [40]	SARPE	N/R	N/R	Dental tipping (100%) Buccal alveolar bone loss (100%)	N/R	N/R
Kayalar E. et al., 2015 [41]	SARPE	N/R	N/R	Dental tipping (100%) Buccal alveolar bone loss (50%) Root resorption (100%)	N/R	N/R
Leyder P. et al., 2018 [42]	SARPE	Insufficient expansion (1.8%) Planned diastema not achieved (3.6%)	20%	Tooth necrosis (3.6%) Gingival recession (3.6%)	Palatal mucosal slough (3.6%)	Screw deformation (3.6%) Osteosynthesis removal (3.6%)
Lim H. et al., 2017 [43]	MARPE	17%	N/R	Dental tipping (100%) Buccal alveolar bone loss (100%)	N/R	N/R
Pereira M.D. et al., 2017 [44]	SARPE	N/R	6%	Tooth discoloration (6%)	Pain (4%) Local infection (2%)	N/R
Ploder O. et al., 2020 [45]	SARPE	N/R	4%	Periodontal attachment loss (4%) Tooth necrosis (4%) Tooth mobility (2%) Root resorption (4%)	N/R	Screw loosening (9%) Screw fracture (4%)
Rachmiel A. et al., 2020 [46]	SARPE	N/R	N/R	Gingival recession (6%) Alveolar bone loss (3%)	N/R	N/R
Sant’Ana L.F.M. et al., 2016 [47]	SARPE	29% (only in the group without midpalatal osteotomy)	N/R	N/R	Discomfort Pain Edema	N/R
Seeberger R. et al., 2015 [48]	SARPE	N/R	N/R	Dental tipping (100%)	N/R	N/R
Sendyk M. et al.,2018 [49]	SARPE	N/R	N/R	Periodontal attachment loss (100%) Gingival recession (100%)	N/R	N/R
Smeets M. et al., 2019 [50]	SARPE	N/R	9%	Bone resorption at midline (3%) Gingival recession (2%) Tooth mobility (2%)	Bleeding (4%) Pain (13%) Neurosensory disturbances (27%) Infection (4%) Lacrimation (1%)	Mechanical failure (3%)
Sygouros A. et al. 2014 [51]	SARPE	N/R	N/R	Dental tipping (100%) Alveolar bending (100%)	N/R	N/R
Wang C. et al., 2023 [52]	MARPE	N/R	N/R	Dental tipping (100%) Alveolar bone loss (100%)	N/R	N/R
Williams B.J.D. et al., 2012 [53]	SARPE	Insufficient expansion (7%)	8%	Tooth discoloration (4%) Gingival recession (10%) Alveolar bone loss (6%) Loss of teeth (2%)	Epistaxis (3%) Hematoma (*n =* 3) Wound infection (7%) Palatal mucosa necrosis (0.8%) Hypoesthesia (3%) Sinus infection (2%) Subcutaneous emphysema (2%)	N/R
Winsauer H. et al., 2021 [54]	MARPE	15%	N/R	N/R	N/R	Screw deformation (15%)
	SARPE	N/R	N/R	N/R	Soft tissue inflammation (3%)	Abutment loss (3%)
Yoon A. et al., 2020 [55]	SARPE	N/R	Minor asymmetric expansion	Tooth necrosis (5%) Periodontal attachment loss (3%)	Paresthesia Dehiscence (3%) Palatal fistula (1%)	N/R

N/R, Not reported.

## Data Availability

The data presented in this study are available in the article.

## References

[1] da Silva Filho O.G., Santamaria M., Capelozza Filho L. (2007). Epidemiology of posterior crossbite in the primary dentition. J. Clin. Pediatr. Dent..

[2] Bucci R., D’Antò V., Rongo R., Valletta R., Martina R., Michelotti A. (2016). Dental and skeletal effects of palatal expansion techniques: A systematic review of the current evidence from systematic reviews and meta-analyses. J. Oral Rehabil..

[3] Smalley W.M., Shapiro P.A., Hohl T.H., Kokich V.G., Brånemark P.I. (1988). Osseointegrated titanium implants for maxillofacial protraction in monkeys. Am. J. Orthod. Dentofac. Orthop..

[4] Landes C.A., Laudemann K., Petruchin O., Mack M.G., Kopp S., Ludwig B., Sader R.A., Seitz O. (2009). Comparison of bipartite versus tripartite osteotomy for maxillary transversal expansion using 3-dimensional preoperative and postexpansion computed tomography data. J. Oral Maxillofac. Surg..

[5] Baysal A., Karadede I., Hekimoglu S., Ucar F., Ozer T., Veli I., Uysal T. (2012). Evaluation of root resorption following rapid maxillary expansion using cone-beam computed tomography. Angle Orthod..

[6] Shayani A., Merino-Gerlach M.A., Garay-Carrasco I.A., Navarro-Cáceres P.E., Sandoval-Vidal H.P. (2023). Midpalatal Suture Maturation Stage in 10- to 25-Year-Olds Using Cone-Beam Computed Tomography-A Cross-Sectional Study. Diagnostics.

[7] Yang P., Zhu M., Guo Y., Su C., Wang Y., Bai Y., Zhang N. (2024). Evaluation of midpalatal suture maturation stage in 5- to 20-year-olds using cone-beam computed tomography. Am. J. Orthod. Dentofac. Orthop..

[8] Angelieri F., Cevidanes L.H., Franchi L., Gonçalves J.R., Benavides E., McNamara J.A. (2013). Midpalatal suture maturation: Classification method for individual assessment before rapid maxillary expansion. Am. J. Orthod. Dentofac. Orthop..

[9] Bays R.A., Greco J.M. (1992). Surgically assisted rapid palatal expansion: An outpatient technique with long-term stability. J. Oral Maxillofac. Surg..

[10] Magnusson A. (2013). Evaluation of surgically assisted rapid maxillary expansion and orthodontic treatment. Effects on dental, skeletal and nasal structures and rhinological findings. Swed. Dent. J. Suppl..

[11] Timms D.J., Vero D. (1981). The relationship of rapid maxillary expansion to surgery with special reference to midpalatal synostosis. Br. J. Oral Surg..

[12] Glassman A.S., Nahigian S.J., Medway J.M., Aronowitz H.I. (1984). Conservative surgical orthodontic adult rapid palatal expansion: Sixteen cases. Am. J. Orthod..

[13] Bell W.H., Epker B.N. (1976). Surgical-orthodontic expansion of the maxilla. Am. J. Orthod..

[14] Morselli P.G. (1997). Surgical maxillary expansion: A new minimally invasive technique. J. Cranio Maxillofac. Surg..

[15] Lindorf H.H., Müller-Herzog R. (2006). Die chirurgisch gesteuerte maxillare expansion (GME) durch selektive Schwachung der Gesichtspfeiler. Zahnheilkd Manag. Kult..

[16] Haas Junior O.L., Matje P.R.B., Rosa B.M., Rojo-Sanchis C., Guijarro-Martínez R., Valls-Ontañón A., Menezes L.M., Hernández-Alfaro F., de Oliveira R.B. (2022). Minimally invasive surgical and miniscrew-assisted rapid palatal expansion (MISMARPE) in adult patients. J. Cranio Maxillofac. Surg..

[17] Mommaerts M.Y. (1999). Transpalatal distraction as a method of maxillary expansion. Br. J. Oral Maxillofac. Surg..

[18] Kraut R.A. (1984). Surgically assisted rapid maxillary expansion by opening the midpalatal suture. J. Oral Maxillofac. Surg..

[19] Messer E.J., Bollinger T.E., Keller J.J. (1979). Surgical-mechanical maxillary expansion. Quintessence Int..

[20] Pogrel M.A., Kaban L.B., Vargervik K., Baumrind S. (1992). Surgically assisted rapid maxillary expansion in adults. Int. J. Adult Orthod. Orthognath. Surg..

[21] Mehra P., Cottrell D.A., Caiazzo A., Lincoln R. (1999). Life-threatening, delayed epistaxis after surgically assisted rapid palatal expansion: A case report. J. Oral Maxillofac. Surg..

[22] Suri L., Taneja P. (2008). Surgically assisted rapid palatal expansion: A literature review. Am. J. Orthod. Dentofac. Orthop..

[23] Benetti M., Montresor L., Cantarella D., Zerman N., Spinas E. (2024). Does Miniscrew-Assisted Rapid Palatal Expansion Influence Upper Airway in Adult Patients? A Scoping Review. Dent. J..

[24] Lee R.J., Moon W., Hong C. (2017). Effects of monocortical and bicortical mini- implant anchorage on bone-borne palatal expansion using finite ele- ment analysis. Am. J. Orthod. Dentofac. Orthop..

[25] Cantarella D., Dominguez-Mompell R., Mallya S.M., Moschik C., Pan H.C., Miller J., Moon W. (2017). Changes in the midpalatal and pterygopalatine sutures induced by micro-implant-supported skeletal expander, analyzed with a novel 3D method based on CBCT imaging. Prog. Orthod..

[26] Choi S.H., Shi K.K., Cha J.Y., Park Y.C., Lee K.J. (2016). Nonsurgical miniscrew-assisted rapid maxillary expansion results in acceptable stability in young adults. Angle Orthod..

[27] Yildirim M., Akin M. (2019). Comparison of root resorption after bone-borne and tooth-borne rapid maxillary expansion evaluated with the use of microtomography. Am. J. Orthod. Dentofac. Orthop..

[28] Tricco A.C., Lillie E., Zarin W., O’Brien K.K., Colquhoun H., Levac D., Moher D., Peters M.D., Horsley T., Weeks L. (2018). PRISMA extension for scoping reviews (PRISMA-ScR): Checklist and explanation. Ann. Intern. Med..

[29] PRISMA. https://www.prisma-statement.org/scoping.

[30] Abate A., Lanteri V., Marcolongo L., Solimei L., Maspero C. (2023). Evaluation of Masticatory Muscles in Adult Patients with Maxillary Hypoplasia Treated with Surgically Assisted Rapid Maxillary Expansion (SARME): A Retrospective Study. J. Clin. Med..

[31] Al-Ouf K., Krenkel C., Hajeer M.Y., Sakka S. (2010). Osteogenic uni- or bilateral form of the guided rapid maxillary expansion. J. Cranio Maxillofac. Surg..

[32] Basu S., Goje S.K. (2023). Comparative Evaluation of Skeletal and Dental Effects of Mini-Implant Assisted and Corticopuncture-Facilitated Rapid Palatal Expansion in Adults: A Randomized Clinical Study. J. Datta Meghe Inst. Med. Sci. Univ..

[33] Choi E.A., Lee K.J., Choi S.H., Jung H.D., Ahn H.J., Deguchi T., Cha J.Y. (2023). Skeletal and dentoalveolar effects of miniscrew-assisted rapid palatal expansion based on the length of the miniscrew: A randomized clinical trial. Angle Orthod..

[34] Contar C.M., Muller P.R., Brunetto A.R., Machado M.A., Rappoport A. (2009). Surgical treatment of maxillary transverse deficiency: Retrospective study of 14 patients. J. Maxillofac. Oral Surg..

[35] Daif E.T. (2014). Segment tilting associated with surgically assisted rapid maxillary expansion. Int. J. Oral Maxillofac. Surg..

[36] Drobyshev A., Klipa I., Drobysheva N., Ilina N., Zhmyrko I. (2021). Surgically Assisted Rapid Maxillary Expansion: Retrospective Analysis of Complications 2012–2017. Georgian Med. News.

[37] Fernández-Sanromán J., Donascimento M.G., López A.C., Ferro M.F., Berrondo I.A. (2010). Transverse maxillary distraction in patients with periodontal pathology or insufficient tooth anchorage using custom-made devices. J. Oral Maxillofac. Surg..

[38] Goldenberg D.C., Alonso N., Goldenberg F.C., Gebrin E.S., Amaral T.S., Scanavini M.A., Ferreira M.C. (2007). Using computed tomography to evaluate maxillary changes after surgically assisted rapid palatal expansion. J. Craniofacial Surg..

[39] Günbay T., Akay M.C., Günbay S., Aras A., Koyuncu B.O., Sezer B. (2008). Transpalatal distraction using bone-borne distractor: Clinical observations and dental and skeletal changes. J. Oral Maxillofac. Surg..

[40] Karabiber G., Yılmaz H.N., Nevzatoğlu Ş., Uğurlu F., Akdoğan T. (2019). Three-dimensional evaluation of surgically assisted asymmetric rapid maxillary expansion. Am. J. Orthod. Dentofac. Orthop..

[41] Kayalar E., Schauseil M., Kuvat S.V., Emekli U., Fıratlı S. (2016). Comparison of tooth-borne and hybrid devices in surgically assisted rapid maxillary expansion: A randomized clinical cone-beam computed tomography study. J. Cranio Maxillofac. Surg..

[42] Leyder P., Altounian G., Quilichini J. (2018). Adjustable selective maxillary expansion combined with one-stage maxillomandibular surgery: A prospective study of osseous widening in fifty-five consecutive patients. J. Cranio Maxillofac. Surg..

[43] Lim H.M., Park Y.C., Lee K.J., Kim K.H., Choi Y.J. (2017). Stability of dental, alveolar, and skeletal changes after miniscrew-assisted rapid palatal expansion. Korean J. Orthod..

[44] Pereira M.D., Koga A.F., Prado G.P.R., Ferreira L.M. (2018). Complications From Surgically Assisted Rapid Maxillary Expansion With HAAS and HYRAX Expanders. J Craniofac. Surg..

[45] Ploder O., Winsauer H., Juengling K., Grill F., Bissinger O., Wolff K.D., Kolk A. (2021). Is There a Significant Difference in Relapse and Complication Rate of Surgically Assisted Rapid Palatal Expansion Using Tooth-Borne, Bone-Borne, and Orthodontic Mini-Implant-Borne Appliances?. J. Oral Maxillofac. Surg..

[46] Rachmiel A., Turgeman S., Shilo D., Emodi O., Aizenbud D. (2020). Surgically Assisted Rapid Palatal Expansion to Correct Maxillary Transverse Deficiency. Ann. Maxillofac. Surg..

[47] Sant’Ana L.F., Pinzan-Vercelino C.R., Gurgel J.A., Carvalho P.S. (2016). Evaluation of surgically assisted rapid maxillary expansion with and without midpalatal split. Int. J. Oral Maxillofac. Surg..

[48] Seeberger R., Abe-Nickler D., Hoffmann J., Kunzmann K., Zingler S. (2015). One-stage tooth-borne distraction versus two stage bone-borne distraction in surgically assisted maxillary expansion (SARME). Oral Surg. Oral Med. Oral Pathol. Oral Radiol..

[49] Sendyk M., Sendyk W.R., Pallos D., Boaro L.C.C., Paiva J.B., Rino Neto J. (2018). Periodontal clinical evaluation before and after surgically assisted rapid maxillary expansion. Dent. Press J. Orthod..

[50] Smeets M., Da Costa Senior O., Eman S., Politis C. (2020). A retrospective analysis of the complication rate after SARPE in 111 cases, and its relationship to patient age at surgery. J. Cranio Maxillofac. Surg..

[51] Sygouros A., Motro M., Ugurlu F., Acar A. (2014). Surgically assisted rapid maxillary expansion: Cone-beam computed tomography evaluation of different surgical techniques and their effects on the maxillary dentoskeletal complex. Am. J. Orthod. Dentofac. Orthop..

[52] Wang C., Liu C., Mao Q., Zhou L., Xiang X. (2023). Skeletal and dentoalveolar modifications in adults with different sagittal facial patterns after personalized miniscrew-assisted rapid palatal expansion: A prospective cone-beam computed tomography study. Am. J. Orthod. Dentofac. Orthop..

[53] Williams B.J., Currimbhoy S., Silva A., O’Ryan F.S. (2012). Complications following surgically assisted rapid palatal expansion: A retrospective cohort study. J. Oral Maxillofac. Surg..

[54] Winsauer H., Walter A., Katsaros C., Ploder O. (2021). Success and complication rate of miniscrew assisted non-surgical palatal expansion in adults-a consecutive study using a novel force-controlled polycyclic activation protocol. Head Face Med..

[55] Yoon A., Guilleminault C., Zaghi S., Liu S.Y. (2020). Distraction Osteogenesis Maxillary Expansion (DOME) for adult obstructive sleep apnea patients with narrow maxilla and nasal floor. Sleep Med..

[56] Ning R., Chen J., Liu S., Lu Y. (2023). Treatment effects after maxillary expansion using tooth-borne vs tissue-borne miniscrew-assisted rapid palatal expansion appliance. Am. J. Orthod. Dentofac. Orthop..

[57] Cozzani M., Nucci L., Lupini D., Tripodi D., Noori N., Hasani M., Jamilian A. (2022). Two different designs of mini-screw assisted maxillary expanders, using FEM to analyse stress distribution in craniofacial structures and anchor teeth. Int. Orthod..

[58] Lin L., Ahn H.W., Kim S.J., Moon S.C., Kim S.H., Nelson G. (2015). Tooth-borne vs bone-borne rapid maxillary expanders in late adolescence. Angle Orthod..

[59] Bi W.G., Li K. (2022). Effectiveness of miniscrew-assisted rapid maxillary expansion: A systematic review and meta-analysis. Clin. Oral Investig..

[60] Alcin R., Malkoç S. (2021). Does mini-implant-supported rapid maxillary expansion cause less root resorption than traditional approaches? A micro-computed tomography study. Korean J. Orthod..

[61] Verdecchia A., Suárez-Fernández C., Miquel A., Bardini G., Spinas E. (2024). Biological Effects of Orthodontic Tooth Movement on the Periodontium in Regenerated Bone Defects: A Scoping Review. Dent. J..

[62] Cantarella D., Dominguez-Mompell R., Moschik C., Mallya S.M., Pan H.C., Alkahtani M.R., Elkenawy I., Moon W. (2018). Midfacial changes in the coronal plane induced by microimplant-supported skeletal expander, studied with cone-beam computed tomography images. Am. J. Orthod Dentofac. Orthop..

[63] Dergin G., Aktop S., Varol A., Ugurlu F., Garip H. (2015). Complications related to surgically assisted rapid palatal expansion. Oral Surg. Oral Med. Oral Pathol. Oral Radiol..

[64] Carvalho P.H.A., Moura L.B., Trento G.S., Holzinger D., Gabrielli M.A.C., Gabrielli M.F.R., Pereira Filho V.A. (2020). Surgically assisted rapid maxillary expansion: A systematic review of complications. Int. J. Oral Maxillofac. Surg..

[65] Kim K.A., Oh S.H., Kim B.H., Kim S.J. (2019). Asymmetric nasomaxillary expansion induced by tooth-bone-borne expander producing differential craniofacial changes. Orthod. Craniofac. Res..

[66] Park J.J., Park Y.C., Lee K.J., Cha J.Y., Tahk J.H., Choi Y.J. (2017). Skeletal and dentoalveolar changes after miniscrew-assisted rapid palatal expansion in young adults: A cone-beam computed tomography study. Korean J. Orthod..

[67] Yoon A., Payne J., Suh H., Phi L., Chan A., Oh H. (2022). A retrospective analysis of the complications associated with miniscrew-assisted rapid palatal expansion. AJO-DO Clin. Companion.

[68] Oliveira C.B., Ayub P., Angelieri F., Murata W.H., Suzuki S.S., Ravelli D.B., Santos-Pinto A. (2021). Evaluation of factors related to the success of miniscrew-assisted rapid palatal expansion. Angle Orthod..

[69] Hamedi Sangsari A., Sadr-Eshkevari P., Al-Dam A., Friedrich R.E., Freymiller E., Rashad A. (2016). Surgically Assisted Rapid Palatomaxillary Expansion With or Without Pterygomaxillary Disjunction: A Systematic Review and Meta-Analysis. J. Oral Maxillofac. Surg..

[70] Bud E.S., Bică C.I., Păcurar M., Vaida P., Vlasa A., Martha K., Bud A. (2021). Observational Study Regarding Possible Side Effects of Miniscrew-Assisted Rapid Palatal Expander (MARPE) with or without the Use of Corticopuncture Therapy. Biology.

[71] Chamberland S. (2023). Maxillary expansion in nongrowing patients. Conventional, surgical, or miniscrew-assisted, an update. J. World Fed. Orthod..

[72] Baik H.S., Kang Y.G., Choi Y.J. (2020). Miniscrew-assisted rapid palatal expansion: A review of recent reports. J. World Fed. Orthod..

[73] Kapetanović A., Odrosslij B.M.M.J., Baan F., Bergé S.J., Noverraz R.R.M., Schols J.G.J.H., Xi T. (2022). Efficacy of Miniscrew-Assisted Rapid Palatal Expansion (MARPE) in late adolescents and adults with the Dutch Maxillary Expansion Device: A prospective clinical cohort study. Clin. Oral Investig..

[74] Peters M.D.J., Godfrey C., McInerney P., Khalil H., Larsen P., Marnie C., Pollock D., Tricco A.C., Munn Z. (2022). Best practice guidance and reporting items for the development of scoping review protocols. JBI Evid. Synth..

